# Hospital Meetings, &c.

**Published:** 1898-03-26

**Authors:** 


### HOSPITAL MEETINGS, &C.

### WEST LONDON HOSPITAL.

The forty-first annual meeting of the West London Hospital was held on March 16th. There was a small attendance. Mr. William Bird, who occupied the chair, said they were opening three wards in the new block, and his fellow members on the board knew that to add 52 beds to those already in use was a bold step in the present state of their finances. The building was finished in May, 1896, and want of funds alone had kept them from opening it before. It was, therefore, necessary that they should do their utmost to get the additional money required. He would only add, since the matter was being mooted, that the question of hospital reform was engaging their attention. The accounts for the year show a total ordinary income of £5,621, against ordinary expenditure of £6,786. The total receipts, including legacies, festival dinner, and building account, were £10,342, and the total expenditure £10,232. The board stated in their report that they learned with astonishment that the Council of the Prince of Wales's Fund limited the "Hospitals of London" to those having more than 100 beds in constant daily occupation. This hospital has a daily average of 91. The board further remarked that out of the sale of Jubilee stamps, which realised £40,000, they had been awarded £525. With regard to the financial position of the hospital, the board regret that in consequence of the insufficiency of the ordinary income to meet the ordinary expenditure and outstanding loans, it was necessary to utilise the proceeds of the festival dinner, as well as the whole of the legacies, instead of investing them as the board would have preferred. The falling off in the number of in-patients was due to the importation of a case of scarlatina into the "Hull" or children's ward. This necessitated the closing of the ward, which included 22 beds, for 44 days. But for this circumstance the in-patients would have numbered at least 50 more. As it was, the total admissions were 1,683, against 1,725 in 1896. After the adoption of the report had been carried, Lord Rothschild was elected to the office of treasurer of the charity.

### ROYAL HOSPITAL FOR DISEASES OF THE CHEST.

The eighty-fourth annual court of governors of the Royal Hospital for Diseases of the Chest took place on Wednesday, the 16th inst., at the hospital, when the Lord Mayor, as usual, presided. Before the meeting was opened the Lord Mayor made a tour of inspection through the hospital. In their report the Council greatly deplored the death of the late President of the hospital, Lord Charles Bruce, and announced that a bed had been dedicated, as a mark of esteem, to his memory. Lord Rothschild had become president in Lord Charles Bruce's place. The Lord Mayor, in moving the adoption of the report and accounts, expressed his regret at finding that there had been a decided falling off in the receipts of the institution for the year 1897, while, on the other hand, there were signs of the fact that an increasing number of people required the attendance which they received in that hospital. It appeared, he said, that there had been a falling off of nearly £4,000 in funds, of which donations accounted for nearly one-half, while the expenditure was some £200 more than last year. He therefore earnestly appealed to the public to remedy that state of affairs. The number of in-patients treated during the year was 740, as compared with 742 in 1896, while the new out-patients numbered 6,356, as compared with 6,583 in the previous year. The motion for the adoption of the report was seconded by the Rev. N. J. Devereux, and was carried unanimously. The election of Lord Rothschild as president of the hospital was confirmed, and votes of thanks to the officials terminated the proceedings.

### DENTAL HOSPITAL OF LONDON.

The Rev. Canon Duckworth presided over the annual meeting of the governors of this institution, held at the hospital on Thursday, March 17th. From the report it appears that the total amount received for the general fund during the year was £2,677, as compared with £2,328 in the previous year, and that the ordinary expenditure amounted to £2,104. The donations of the life governors were £552, against £368 in 1896, while the general donations amounted to £42, as against £36. The annual subscriptions, totalling £1,226, show the trifling decrease of £19. In reference to this the report remarks, "The most notable and least satisfactory point in the foregoing statement is the falling off in annual subscribers. This we must take in common with almost every similar institution as the result of unusual circumstances; for not only has the tide of our social life been disturbed in its ebb and flow, but the ordinary current of charity was broken up by new and extraordinary demands, both local and general." The amount received from the Prince of Wales's Fund was £109, and, dealing with the question, Mr. F. A. Bevan, in moving the adoption of the report, observed that he feared that fund, instead of helping the hospitals, would have the reverse effect. He had heard of a case of an eminent firm who, on being applied to for subscriptions they had been in the habit of giving, stated that they had put down their names for a certain sum for the Prince of Wales's Fund, and intended to give no more. In reference to the fund for building the new hospital, the report stated that the total amount contributed during the year was £2,928. The estimates for the hospital have been received, and are now under consideration of the trustees, and the committee look forward to the immediate commencement of building work. The report of the Medical Committee shows that the number of operations advanced from 57,654 in 1896 to 62,512 in 1897, an increase of nearly 5,000 on those of the preceding year, and 43,257 in excess of 1874, when the hospital was moved to the present buildings. These included 31,851 extractions, 20,274 of which were under nitrous oxide. The committee report that, it having been found convenient for certain students to obtain their mechanical training within the hospital rather than in the workroom of a private practitioner, it has been decided to admit a limited number of pupils to the school for this purpose. The formal resolutions were unanimously adopted.

### UNIVERSITY COLLEGE HOSPITAL.

Lord Monkswell, who presided at the annual meeting of this hospital on Friday, 18th inst., expressed great regret at the want of success which had attended the efforts of the local committee appointed last year to stir up interest in the immediate district. Apart from a donation of £250 from the Duke of Bedford—who has accepted the office of president—the committee had only raised £343, a result which appeared to show lamentable local indifference to the existence of the charity. Donations and subscriptions generally showed an improvement of over £1,000 on the previous year, and the festival dinner—the first at which ladies had attended—realised £5,309, a record amount. Legacies, on the other hand, showed a serious falling off, only £722 available for general purposes having been received last year as against £3,457 for 1896, and an average of £6,689 for the past ten years. In consequence of the failure of the efforts to obtain increased support, the committee were compelled early in December to close fifty out of the 210 beds. Shortly afterwards, however, a cheque for £2,581 was received from the Prince of Wales's Fund. Of this sum £1,181 was a special donation for 1897 only, while £1,400 was allotted to encourage the committee to keep open at least twenty-five of the recently closed beds, the continuance of the subscription being subject to the condition that it be devoted to that purpose. Steps were at once taken to reopen twenty-five beds, the total number now available being 185. Referring to Sir J. Blundell Maple's munificent offer of about £20,000 for rebuilding the hospital, the committee were glad to report that the negotiations for the property at the rear of the hospital had been brought to a satisfactory issue, and the building was now well in hand. It was necessary, however, to emphasise the fact that Sir Blundell's gift was for building purposes only, and would not place the committee in a better position as regards the maintenance of the institution. Mr. Henry Lucas, the chairman of the Hospital Committee, who also dealt with the financial position, asserted that unless some unexpected source of income arose they would either have a very large debt at the end of the year or be compelled to close more beds. During 1897 the in-patients numbered 2,875, the total cost per occupied bed being £79 8s. The out-patients, including 1,952 lying-in cases, numbered 14,658, and there were also 35,419 casualties. The report was unanimously adopted.

### ST. MARY'S HOSPITAL.

Colonel Stanley G. Bird, who presided at the annual meeting of St. Mary's Hospital, on Friday, March 18th, had a most favourable statement to present to the governors. The report of the Board of Management states that 1897 was financially one of the most satisfactory in the history of the hospital. The legacies amounted to no less than £17,689, and enabled the board to replace most of the capital sold to provide for current expenditure in the unprosperous years 1893-6. There was also an increase in annual subscriptions, raising the receipts from that source to £5,380, the highest figures yet attained, and £7,250 was received for the endowment of beds. The income apart from the endowment donations amounted to £32,924, and the expenditure to £21,815. With regard to the Prince of Wales's Fund from which St. Mary's received in all £2,706, the board remark that if this fund prospers, as all hope, it will confer great and lasting benefits on the hospitals. These advantages will not be confined to the direct pecuniary aid brought to the hospitals by the fund, but by the system of inspection which the Council has decided to make the basis of its awards, public confidence in the hospitals will be greatly strengthened and the amount of support bestowed upon them be correspondingly increased. The board therefore greet with satisfaction the establishment of the fund, and express their thanks to His Royal Highness for the great service he has rendered to the hospitals by its establishment. The in-patients treated at the hospital during 1897 numbered 3,317, as against 3,711 in the year preceding, the decrease being due to the fact that in 1897 the hospital was closed for two months for repairs. The daily average number resident was 200, the mean residence of each patient 21 days, and the rate of mortality 8·02 per cent. There were 17,309 out-patients, 17,430 casualties, and 1,596 maternity patients. The report further states that a casualty physician, in the person of Dr. John F. H. Broadbent, has been appointed with the view of restricting the number of cases to be seen each day by the out patient physician and eliminating trivial cases. An inquiry officer has also been engaged with the object of preventing abuse of the charity by those able to pay for medical treatment. With regard to the addition to the hospital, considerable progress has been made with the new wing, and the new out-patient department, which is the most costly and complicated part of it, will shortly be ready for occupation. Dr. Sidney Phillips, in responding to a vote of thanks to the medical and surgical staff, stated that, owing to the want of the new wing, they had discharged their duties under great difficulties, and when that wing was opened it would prove a great advantage to the hospital.

### THE HAMPSTEAD HOSPITAL.

The annual meeting of governors of the Hampstead Hospital was held at the Vestry Hall, Hampstead, on Saturday, March 19th, under the presidency of Mr. E. Bond, M.P., L.C.C. The Chairman congratulated the meeting on the fact that for the first time in the history of the hospital they commenced the year with a balance on the right side. The ordinary income amounted to £2,133, and in addition the sum of £1,500 was received from the Hampstead Diamond Jubilee Fund, making a total of £3,633. The expenditure amounted to £2,873, and after paying off the overdraft at the bank the council had in hand a balance of £170. The Ladies' Association has rendered substantial assistance in the direction of raising funds, and the council believe that the efforts of the ladies during the current year will yield still greater results. It was originally intended that this hospital should be used solely for the benefit of paying patients, but the idea has been departed from, and out of the 305 in-patients admitted during 1897 231 received free treatment, while 52 paid 12s. per week, and 22 from £2 2s. to £5 5s. per week. In addition to these there were 201 minor casualties and accidents, but it was not necessary that the patients concerned should stay in the hospital more than one day. The cases in the out-patient department numbered 1,610. In the report of the medical staff, which was read by Dr. Ware, the desirability of appointing a resident officer to deal with urgent cases was referred to, and the staff further expressed the hope that the increased prosperity of the institution would result in the erection of a new and more suitable hospital building. In connection with the latter point it was subsequently mentioned that consequent on the efforts of the Ladies' Association, the council had a special sum of £100 in hand, which would form the nucleus of a building fund. The council recognise the importance of both recommendations, but state that the financial difficulty stands in the way at present. A noticeable feature in this hospital's statement of accounts is the cost of administration, which for 1897 amounted only to £117.

### CHELSEA HOSPITAL FOR WOMEN.

The twenty-seventh annual general meeting of this institution was held at the hospital on Monday, March 21st. Lord Glenesk, who presided, congratulated the meeting on the fact that the report was of a most encouraging character. The in-patients numbered 693, against 596 in the year preceding, and there were 175 major operations, as against 109 in 1896. The average number of in-patients per day was 44·9, and the mortality for the year, based on the number of patients discharged, including those who died, 2·3. The Council believe that the remarkable increase in the number of critical cases has probably resulted from the publication of the excellent record of 1896. There were 2,619 new cases in the out-patient department during the year, and it may be mentioned that on the out-patient letters a notice has been printed urging upon the subscribers the importance of not granting them to "persons who appear to be in a position to obtain medical aid without attendance at a hospital." The Council trust that the carrying out of this recommendation, coupled with the efforts made at the hospital, will have a salutary effect. Of the 693 in-patients 491 are described as housewives, 65 as of no occupation, 51 as cooks and domestics, 13 as hospital nurses, and 12 as governesses. Though no legacies were received during 1897, the statement of accounts is of a very satisfactory nature. The total income, including £306 from the Prince of Wales's Fund and £356 brought forward from the previous year, amounted to £5,680. The total expenditure was £4,415, so that at the close of 1897 there was a balance of £1,265 in hand. Lord Glenesk referred with satisfaction to the increased grants received from the Hospital Sunday and Saturday Funds, and paid a warm tribute to the medical staff and to Miss M. Heather Bigg, and the nurses under her charge. It has been decided to hold a festival dinner this year on June 16th, and the idea has already received warm support in influential quarters. Lady Dufferin, in writing to express her willingness to become a patron of the dinner, remarked upon the help and instruction students from India had received at the hands of the hospital authorities. The work of the Convalescent Home at St. Leonards has materially increased during the year, the number of patients reaching a total of 231, as compared with 194 during 1896. As a result of this increase the expenditure exceeded the income by nearly £70. Mr. H. E. Wright seconded the adoption of the report, which was carried unanimously. A vote of thanks to the honorary officers concluded the proceedings.

### EAST LONDON HOSPITAL FOR CHILDREN.

The festival dinner of the East London Hospital for Children was held on Tuesday, the 22nd inst., at the Hotel Metropole. Those present included Mr. Benjamin L. Cohen, M.P., who presided, and Mrs. Cohen, Viscount and Viscountess Falkland, Sir Henry Burdett, K.C.B., the Hon. Alban Gibbs, M.P., Mr. Harry S. Samuel, M.P., and Mr. H. W. Trinder.

The Chairman, in proposing "The Queen," said this toast would be drunk with the more enthusiasm because Her Majesty was a patron of the Hospital, and was one of the most munificent of their annual subscribers. (Applause.) In proposing the toast of "The Prince and Princess of Wales and the rest of the Royal Family," he said that the management of the hospital were most grateful for the donation of nearly £400 which they had received from the Prince of Wales' Hospital Fund. The gratitude was perhaps a little mixed with the hope of favours, and he might say, in the presence of Sir Henry Burdett, of possibly increased favours to come. (Applause.)

With reference to the toast of the evening, "The East London Hospital for Children," the Chairman confessed that when he reflected on the extent that this hospital was dependent for its efficiency, nay, almost for its existence, on the success of its festival dinner, he was a little appalled at the responsibility which devolved on him. It was not necessary for him to say much as to the character and the needs of the hospital. Its very name pleaded for it more than he could, as it told its own tale. It was for children who were helpless, and could not plead for themselves, and it was in the East of London, where the parents were almost in the same helpless and hopeless condition as that in which their children were placed.

Mr. H. W. Trinder, Chairman of the Board of Management, responded. He said that during the thirty years of its existence the hospital, besides carrying on its ordinary work, had spent about £40,000 on rebuilding and new buildings, and had invested about £25,000, which, though a large sum, was some way off the five years' income which it was held every well administered hospital should have put away in order to meet the bad years which every institution must have to face.

Sir Henry Burdett, in proposing the health of "The Board of Management," said he was glad to see from the report that the management of the hospital recognised that the grant received from the Prince of Wales's Fund was given with a due sense of responsibility, and with the warm desire to help as far as possible, even in the first year of the Fund's existence, all the hospitals in London. Speaking on the general question of the hospitals of London so far as the Prince of Wales's Fund was concerned, he said the objects of the Prince of Wales's Fund had been very much misunderstood. They would remember that the Prince of Wales set out to raise £100,000 per annum for the hospitals of London, and the result of his appeal was this. In the first year a sum not far short of a quarter of a million had been raised, and the sum actually distributed was the sum which was necessary to meet the requirements of all hospitals whose needs there was time to investigate in the limited time at the Council's disposal, and to make a bonus gift equivalent to that of the Hospital Sunday Fund to all the hospitals which received a grant from that Fund. The Council of the Prince of Wales's Fund had also been able to establish their position by setting aside a certain amount to meet contingencies. The criticisms raised against the Prince of Wales's Fund by certain people, was that it had not been of much benefit to the hospitals of London, nay more, that it had been a positive evil. For twenty-five years he had been working for hospitals, and the same old cry had been levelled against every new movement which had been made on behalf of the hospitals. That cry had been levelled at the Hospital Sunday Fund, in fact, even now it was sometimes heard, despite the evidence to the contrary. He had taken the trouble to test the statement that the London hospitals had suffered financially during the year of Diamond Jubilee, and he was glad to say that he had found, that so far from this being the case, it was distinctly opposed to the truth. Four of the most important hospitals were able to report that the Diamond Jubilee year was the most prosperous year in their history, and if they took all the hospitals which could be called metropolitan hospitals, it would be found that out of the whole eighty-three there were only about six, and these six were insignificant ones, where the receipts had not been equal to the average of former years. He could, if he chose, give the reasons why these six institutions had not prospered like the majority of their fellows, but that was not the place to indulge in criticism of the kind. Every hospital which had not gone to sleep, but had put forth energetic efforts to secure the necessary funds during the Diamond Jubilee year had reason to be satisfied with the results. The few who suffered might be roughly described as the foolish virgins of the hospital world. As to the East London Hospital, he bore testimony to its excellent management, saying that it was conducted at a cost which was something like £10 per bed cheaper than that of any other metropolitan hospital for children. The efficiency of every department was at the same time undoubted. (Applause.)

Viscount Falkland replied.

Other toasts followed. The collection amounted to £1,745.

### ROYAL LONDON OPHTHALMIC HOSPITAL.

Sir John Lubbock, M.P., presided at the annual meeting of governors held on Tuesday, 22nd inst. There were also present Messrs. F. H. Janson, Mr. H. P. Sturgis, Mr. E. Hogg, and other leading supporters of the institution. The accounts for 1897 show a total income of £5,072, whilst the expenditure amounted to £6,379, leaving a deficiency of £1,307. The Chairman, in referring to the financial position, said he wished to emphasise the fact that, but for the legacies they had received, the balance on the wrong side would have amounted to £2,681. The committee declare that they cannot see their way to reduce the annual expenditure without impairing the efficiency of the hospital. The alternative course is to curtail the work, and, in face of strong evidence of the usefulness of the charity, they are reluctant to take such a step without first making a strenuous effort to obtain more regular support by means of annual subscriptions. With this end in view, they have appointed a special appeal committee, and H.R.H. the Duke of Cambridge has consented to take the chair at a festival dinner on May 6th. The committee point out that the hospital is absolutely free to poor persons suffering from diseases of the eyes, that no letters of recommendation are required, and further, that the institution gives spectacles to patients who are unable to to buy them, and help others by paying part of the cost. The total number of in-patients during the year was 1,968, the out-patients numbering 25,051, and their total attendances 129,950. The inquiry officer, appointed some years since for the purpose of preventing any abuse of the charity, was obliged during 1897 to refuse 602 applicants, and these, in nearly every case, admitted they were able to pay a surgeon, but stated they were not aware that this hospital was open only to the poor. The new hospital buildings in the City Road are rapidly approaching completion, and it is hoped that they may be occupied in the course of the next twelve months.

### THE POPLAR HOSPITAL.

The Hon. Sydney Holland presided at the forty-third annual meeting of the Poplar Hospital for Accidents, held on Tuesday, March 22nd. The Chairman remarked that he must first draw attention to one unsatisfactory point in the report before them. This was the falling off in the donations, which showed a decrease of £3,600 as compared with 1896. This was a serious matter for a small hospital, since it was essential they should have an adequate reserve against bad times. At present their reserve fund was a small one, and it ought to be equivalent to five years' annual income. This, according to so high an authority as Sir Henry Burdett, was a safe amount for a hospital to have in hand. Mr. Holland proceeded to point out that, while the donations showed a decrease, there was a considerable increase in annual subscriptions; in fact, within the last five years the income from this source had more than doubled. Another satisfactory feature was the support given to the hospital by working men. The amount subscribed direct to the hospitals of London by working men was £5,000 per annum, and out of that £570 was given to the Poplar Hospital by the men of the district. The total receipts for the year, including £7,155 brought forward from 1896, this amount being intended to go towards the liability of the building fund, was £15,351, and the expenditure £11,771. The committee, however, point out that of the credit balance of £3,580, £3,200 is required to meet the building and architects' accounts. They also observe that they do not think that any of the falling off in the donations this year is traceable to the operation of the Prince of Wales's Fund. Dealing with the work of the hospital, the Chairman pointed out that 1897 was the record year of the institution. The in-patients numbered 1,037, against 876 in the previous year, and the out-patients 19,045, as against 17,651. This proved that the hospital was needed, and, as a matter of fact, it was doing splendid work. It appears from the report and from the chairman's remarks that the past year has been an eventful one in the history of the hospital. In July the new operating theatre was opened, and also the new receiving-room; while September saw the opening of the isolation block, and this has been of immense service in preventing infection spreading through the wards on twelve different occasions. By the adoption of Mr. Holland's "Charter of Liberty for Nurses," the nursing staff, in addition to two hours off every day, get a half-day off every week, a whole day every month, and four weeks' holiday every year. Mr. Holland referred in high terms to the nurses of the hospital, and several working men representatives at the meeting also spoke most gratefully of the kindness and devotion displayed by the matron and her staff. As regards the future, the report states that all the hospital needs in the way of capital expenditure are sitting-rooms for the three ward sisters, built off the wards. These will cost about £1,200, and the committee are further considering the advisability of building a laundry. The adoption of the report and other formal resolutions was unanimously carried.

WORKING MEN AND HOSPITALS.

The second of the series of meetings arranged by a joint committee of the Federation of Working Men's Social Clubs and the Club and Institute Union was held on Wednesday, the 16th inst., Mr. Harold Boulton in the chair. The subject before the meeting was: "To consider what (if any) reforms are requisite in the out-patients department." Amongst the speakers were the Hon. Sydney Holland, a representative from Guy's Hospital, and Dr. Davis, of the Metropolitan Hospital.

